# Effectiveness of a Smartphone Application for Dietary Sodium Intake Measurement

**DOI:** 10.3390/nu15163590

**Published:** 2023-08-16

**Authors:** Chan-Young Jung, Youngin Kim, Hyung Woo Kim, Seung Hyeok Han, Tae-Hyun Yoo, Shin-Wook Kang, Jung Tak Park

**Affiliations:** 1 Department of Internal Medicine, College of Medicine, Yonsei University, Seoul 03722, Republic of Korea; cyjung91@gmail.com (C.-Y.J.);; 2 Division of Nephrology, Department of Internal Medicine, Asan Medical Center, Seoul 05505, Republic of Korea; 3 Noom, Inc., Seoul 07327, Republic of Korea; 4 Institute of Kidney Disease Research, Yonsei University, Seoul 03722, Republic of Korea

**Keywords:** application, smartphone, sodium intake, technology, urinary sodium

## Abstract

Accurate estimation of sodium intake is a key requirement for evaluating the efficacy of interventional strategies to reduce salt intake. The effectiveness of a smartphone application in measuring dietary sodium intake was assessed. This study included 46 participants who consented to register in Noom’s food-logging program. All participants were followed up for six months from the day of enrollment. The mean age of the participants was 40.2 ± 12.3 years, and 22 (48%) participants were male. The average number of times/weeks the meals were logged was 16.2 ± 10.3. At baseline, the mean 24-h urine sodium was 124.3 mmol/24 h. The mean sodium intake measured by the smartphone application and calculated using the 24-h urine sodium was 2020.9 mg/24 h and 2857.6 mg/24 h, respectively. During the second visit, the mean 24-h urine sodium was 117.4 mmol/24 h. The mean sodium intake measured by the smartphone application and calculated using the 24-h urine sodium was 1456.0 mg/24 h and 2698.3 mg/24 h, respectively. Sodium intake measured using the smartphone application positively correlated with that calculated using the 24-h urine sodium at baseline (*r* = 0.464; *p* < 0.001) and follow-up (*r* = 0.334; *p*= 0.023). Dietary sodium intake measured using a smartphone application correlated well with that estimated using 24-h urine sodium level.

## 1. Introduction

High salt intake, primarily by means of raising blood pressure [1], is associated with significant cardiovascular morbidities and mortality [2,3]. The average daily salt intake of the general population is estimated to be between 10–15 g [4,5,6], which far exceeds both physiological requirements and the World Health Organization target of <5 g NaCl/day [7]. Recent studies have indicated that reducing salt intake could substantially reduce the risk of cardiovascular morbidities and mortality [8,9].

Considering the adverse outcomes of excess salt intake, several studies have investigated effective salt-reduction strategies ranging from population to individual-level interventions [10]. For instance, the United Kingdom successfully implemented a salt reduction program in 2003, resulting in a 15% reduction in 24-h urinary sodium for seven years [11]. In another randomized controlled trial involving participants with chronic kidney disease, a web-based coaching intervention was employed. This intervention encompassed self-monitoring aided by a comprehensive interactive food diary, effectively lowering sodium intake within three months [12]. Nevertheless, it is important to acknowledge the likelihood of under-reporting when utilizing food frequency questionnaires (FFQs) or dietary recall methods [13,14,15]. Therefore, the findings from these salt-related studies should be approached with caution. While the accurate assessment of dietary sodium intake remains pivotal for evaluating the effectiveness of these interventional approaches, it is worth noting that no standardized, universally accepted methodology currently exists.

As a newly emerging technology, investigations have been conducted to determine whether smartphone application-based dietary assessment improves dietary collection than the conventional methods [16,17,18]. Several studies have indicated that smartphone applications significantly correlate with FFQs in the estimation of energy intake, macro- and micronutrients, and alcohol consumption [17,19]. However, whether dietary data collected using smartphone applications correlates well with sodium intake has not been previously investigated.

In this study, the effectiveness of a smartphone application in measuring dietary sodium intake was assessed. This was done by collecting dietary data using a smartphone application and correlating these findings with laboratory data.

## 2. Methods

### 2.1. Study Design and Participants

**Figure 1:**
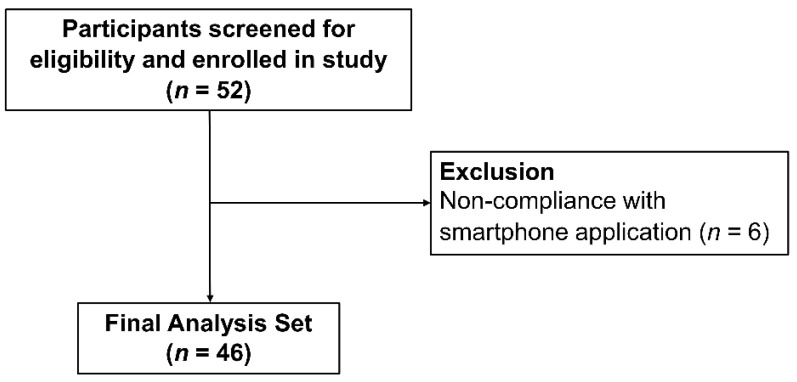
Flow diagram of the study.

This prospective study assessed patients who underwent regular follow-up at the Nephrology clinic of Severance Hospital, Yonsei University Health System (YUHS), Seoul, Republic of Korea. Those who met the following criteria were excluded: (1) age < 18 years, (2) baseline estimated glomerular filtration rate (eGFR) < 30 mL/min/1.73 m^2^, and (3) application non-compliance, which was defined as meals logged less than three times/week (Figure 1). Each participant provided written consent to participate in the study and registered for Noom’s food logging program. The participants were followed up for six months from the day of enrollment. The present study was conducted in accordance with the Declaration of Helsinki and was approved by the Institutional Review Board of YUHS (4-2017-0906).

### 2.2. Smartphone Application-Based Dietary Assessment

**Figure 2:**
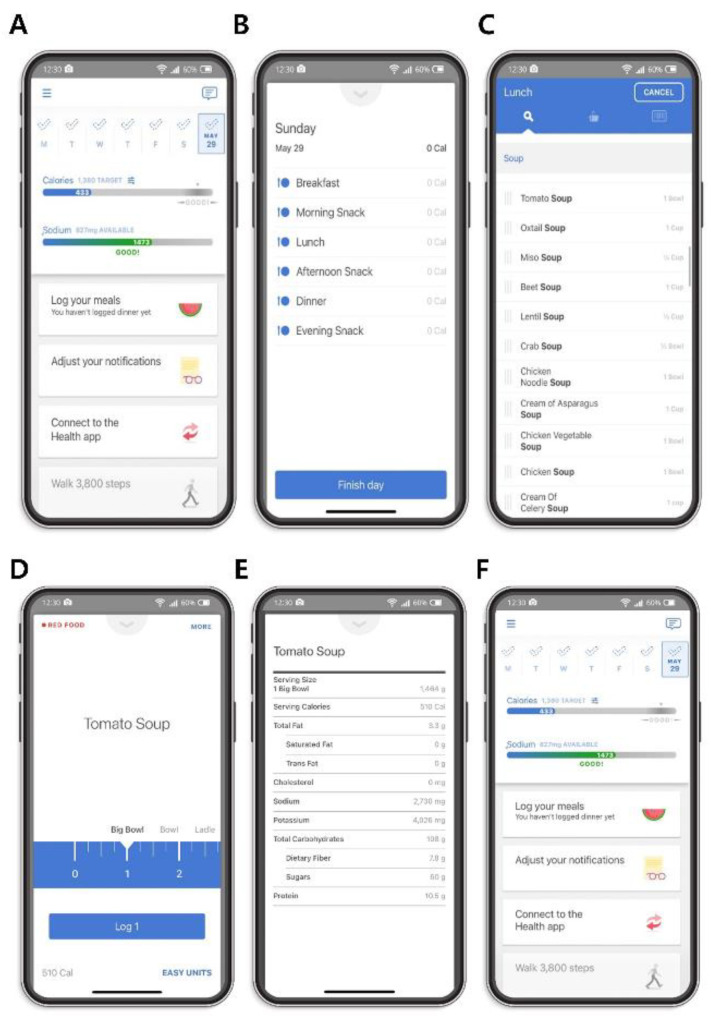
Features of the Noom Health application. (**A**) Participants were instructed to log their meals throughout the day using the smartphone application by selecting the ‘Log your meals’ icon, where participants first selected (**B**) which meal (breakfast, morning snack, lunch, afternoon snack, dinner, or evening snack) they were logging. (**C**) Participants then logged the type of meal by typing their meal in the grey search box (**D**) and selected the amount and units of food they had consumed by swiping their fingers across the blue tape measure and the units tab above, respectively. (**E**) Participants could also view key nutritional facts (serving calories, total fat, saturated fat, trans fat, cholesterol, sodium, potassium, total carbohydrates, dietary fiber, sugars, and protein) related to the selected food (**F**). They were also able to monitor their daily calorie and sodium intake throughout the day.

All participants were instructed to use Noom Health’s food logging feature and the group and private message functions during the 6-month study period free of charge (Figure 2). The Noom Health application (Noom, Inc., Seoul, Republic of Korea) is a diet self-management smartphone application commercially available from Google’s Play Store and Apple’s App Store. Human coaches, who are qualified clinical dietitians, assist application users online as part of the application’s features. The application allows individuals to self-monitor their food intake using reminders.

Before study initiation, all participants were given detailed instructions on how to use the smartphone application. They were instructed to log their meals throughout the day using the smartphone application by selecting the ‘Log your meals’ task card on the smartphone application’s interface (Figure 2A). This action brought up a pop-up menu where participants could select which meal (breakfast, morning snack, lunch, afternoon snack, dinner, or evening snack) they were logging (Figure 2B). Participants then entered the type of meal they had consumed in the grey search box at the top of the screen (Figure 2C). For example, if the participant had consumed soup for breakfast, typing ‘soup’ in the search box brought up the many possible types of soup consumed. Selecting the specific type of meal that the participants had consumed brought up a pop-up screen that enabled them to select the amount of food they had consumed. If the participants had consumed soup, they could then select how much of it they had consumed in units of bowls by swiping their fingers across the tape measure to select the correct portion size (Figure 2D). If the type of food consumed was not measurable in the form of bowls, they had the option of selecting from a list of other commonly used measurements, including servings, and cups, by swiping their fingers across the units of measure at the top of the tape measure to select a different unit of measure (Figure 2D). The application did not necessitate input of food weights. Upon selecting the type and amount of meal consumed, the Noom application also allowed participants to look up nutritional facts that included the number of calories, total saturated and trans-fat, cholesterol, sodium, potassium, total carbohydrates, dietary fiber, sugars, and total protein related to that particular food (Figure 2E), by selecting ‘More’ on the upper right-hand corner of the food selection screen (Figure 2D). The Noom Health application utilizes the Open-Source Food Safety Database from the Korean Ministry of Food and Drug Safety [20], the Korean Standard Food Composition Table, which is updated every five years by the Korean Rural Development Administration National Institute of Agricultural Sciences [21], and the Computer Aided Nutritional analysis program provided by the Korean Nutrition Society when calculating the nutritional information entered by the application users [22]. The Noom application also uses a color-coding system to categorize foods in the form of red, green, and yellow color system (Figure 2D). Green foods indicate the least calorie-dense foods or contain the highest concentration of healthy nutrients, such as vegetables and whole grains, or both. Yellow foods have more calories or less healthy nutrients per serving than green foods, such as lean meats, starches, or both. Red foods are the most calorie-dense or have the least healthy nutrients, such as red meats, desserts, or both [23]. For example, tomato soup was categorized as a red food (Figure 2D).

Once the participants logged their meals into the Noom application by selecting ‘Log’ (Figure 2D), they could view the number of calories and sodium consumed throughout the day and the number of remaining calories and sodium to meet the participant’s daily calories and sodium goal. The participant’s daily calorie target minimum was calculated based on a number of factors. Specifically, Noom used the basic principles of the Harris-Benedict equation to establish the participant’s minimal daily calorie intake [24]. Using this principle, the participant’s basal metabolic rate, which was based on the participant’s sex, age, height, and starting weight, was determined. The participant’s basal metabolic rate was then applied to the Harris-Benedict equation to determine the participant’s daily calorie target. Although Noom assumes a sedentary lifestyle for each participant, participants can increase that total calorie target allowance for that day by logging exercises throughout the day (not shown). The participant’s daily sodium target was determined according to the guidelines of the Dietary Approaches to Stop Hypertension (DASH) diet, which recommends a sodium intake of 2300 milligrams [25]. The participant’s daily calorie and sodium intake was calculated based on the types of meals logged in the smartphone application throughout the day (Figure 2F). Sodium intake during the first visit (baseline) and second visit (6 months after the first visit) was measured by averaging the daily sodium intake during the first and last weeks of application usage, respectively. Notably, the study duration (June 2018–February 2020) did not encompass the availability of the option in the latest version of the Noom Health application, allowing users to log meals by scanning barcode labels using smartphone cameras.

To encourage active application usage, participants were given instructions on interacting with their respective human coaches through the smartphone application. The human coaches communicated with the participants regularly via in-application private and group messages. Participants regularly received messages that encouraged regular meal logging. Application usage was assessed based on the number of meals logged per week. Participants were sent reminder messages if they had not logged any meals for more than three days.

### 2.3. Data Collection and Measurements

Demographic, anthropometric, and laboratory data were collected during enrollment. Baseline demographic and anthropometric data included age, sex, blood pressure, height, weight, past medical history, and current medications. Body mass index (BMI; kg/m^2^) was calculated as the body weight divided by the height squared. Baseline blood chemistry data collected during enrollment included urea nitrogen, creatinine, glucose, cholesterol, albumin, uric acid, serum sodium, and serum potassium. Serum creatinine level was measured using the isotope dilution mass spectroscopy-traceable method at a central laboratory, with calibration against the reference. The eGFR was calculated using the Chronic Kidney Disease Epidemiology Collaboration (CKD-EPI) creatinine equation [26]. Urinalysis was performed at baseline and six months after enrollment. Urinalysis data included random urine sodium, 24-h urine sodium, random urine albumin, 24-h urine albumin, and random urine creatinine. The 24-h urine collection was complete if the collected urine volume was ≥800 mL. Based on the 24-h urine sodium level, the sodium intake was estimated by first calculating the estimated daily NaCl (g/24 h) intake from the 24-h urine sodium excretion [27] and then converting this to the estimated daily sodium intake using the following formula [28]:

Daily sodium intake (mg) = daily NaCl intake (g/24 h) × 393.7.

### 2.4. Statistical Analyses

Continuous variables are expressed as means and standard deviations or medians and interquartile ranges, and categorical variables are expressed as numbers and percentages. Differences in the sodium intake measured by the smartphone application and calculated based on the 24-h urine sodium level were evaluated using Student’s *t*-test. Univariate correlations between the measured and calculated sodium intakes were analyzed using Pearson’s correlation. Moreover, the Bland-Altman method was used to measure the agreement between the measured and calculated values. Statistical significance was defined as *p* < 0.05. Data were analyzed using STATA version 15 (STATA Corp., College Station, TX, USA).

## 3. Results

### 3.1. Baseline Characteristics

A total of 46 participants were included in the final analysis (Figure 1). The baseline characteristics of the study participants are presented in Table 1. The mean age was 40.2 years, and 22 (48.0%) participants were male. The mean BMI was 23.8 kg/m^2^. All participants had hypertension at baseline and were all taking anti-hypertensive agents. Among them, 11 (24.0%) participants were concomitantly on anti-dyslipidemic agents. The mean serum creatinine was 1.0 mg/dL, and the mean eGFR was 86.5 mL/min/1.73 m^2^. At baseline, the mean random urine sodium level was 88.1 mmol/L, and the mean 24-h urine sodium level was 124.3 mmol/24 h. The median albumin-to-creatinine ratio was 287.1 mg/g of creatinine, and the median 24-h urine albumin level was 229.6 mg/24 h. The mean number of times/week that the meals were logged was 16.2.

### 3.2. Difference between Sodium Intake Measured by the Application and Calculated Using 24-h Urine Sodium Level

At baseline, the mean 24-h urine sodium level was 124.3 mmol/24 h. The mean sodium intake measured by the smartphone application and calculated based on the 24-h urine sodium level was 2020.9 mg/24 h and 2857.6 mg/24 h, respectively. During the second visit, the mean 24-h urine sodium level was 117.4 mmol/24 h. The mean sodium intake measured by the application and calculated based on the 24-h urine sodium level was 1456.0 mg/24 h and 2698.3 mg/24 h, respectively (Table 2). The differences between the sodium intake measured by the application and that calculated using the 24-h urine sodium level during both visits were all statistically significant (*p* < 0.001).

### 3.3. Correlation between Sodium Intake Measured by the Application and That Calculated Using 24-h Urine Sodium Level

**Figure 3:**
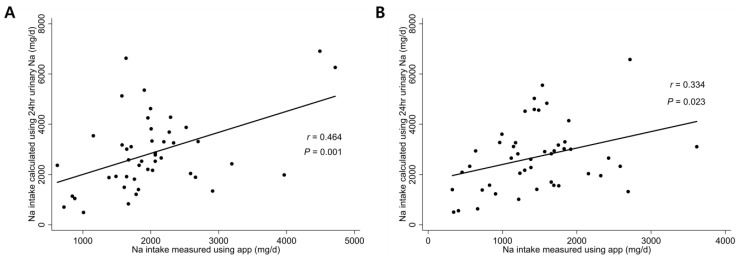
Correlation between dietary sodium intake measured by the smartphone application and that calculated using 24-h urine sodium at (**A**) baseline and (**B**) 6 months after enrollment.

Figure 3 shows the univariate correlations between sodium intake measured by the smartphone application and that calculated based on the 24-h urine sodium level at baseline and follow-up. Sodium intake measured using the smartphone application correlated positively with that calculated using the 24-h urine sodium level at baseline (*r* = 0.464, *p* < 0.001; Figure 3A) and follow-up (*r* = 0.334; *p* = 0.023; Figure 3B).

**Figure 4:**
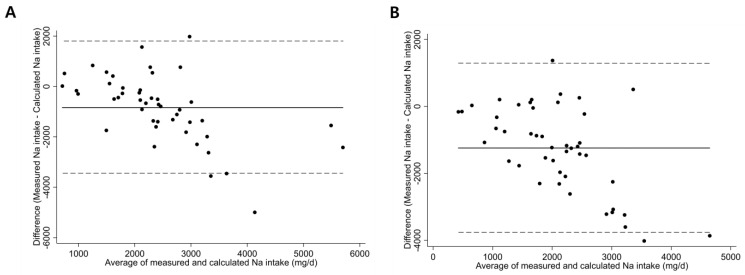
Bland-Altman plots showing mean difference and limits of agreement between dietary sodium intake measured by the smartphone application and that calculated using 24-h urine sodium at (**A**) baseline and (**B**) 6 months after enrollment. (**A**) Mean difference = −836.8, limits of agreement = −70.3, 1797.0, (B) Mean difference = −24.1, limits of agreement = −76.3, 4646.4.

The Bland-Altman plots in Figure 4 demonstrate that the correlation between sodium intake measured by the smartphone application and that calculated using the 24-h urine sodium level was reasonably uniform across all data ranges. The mean difference (95% limits of agreement) between sodium intake measured by the smartphone application and that calculated using the 24-h urine sodium level at baseline (Figure 4A) and follow-up (Figure 4B) was -836.8 mg (−70.3 mg to 1797.0 mg) and -24.1 mg (−76.3 mg to 4646.4 mg), respectively.

## 4. Discussion

In this study, the dietary data collected using a smartphone application correlated well with dietary sodium intake estimated from laboratory data obtained from the study participants. Although the dietary sodium intake estimated using the smartphone application was lower than that calculated using laboratory data, the correlation between these two methods of estimation was reasonably uniform across all ranges of data. Due to the limited availability of clinical data exploring the utilization of smartphone applications for measuring dietary sodium intake, the outcomes of this study lend additional support to the need for continued investigation of smartphone applications’ efficacy in measuring dietary sodium intake.

The findings of this study suggest the potential benefits of using smartphone applications to assess dietary sodium intake. Considering that dietary sodium intake strongly associates with adverse health outcomes [8,9], accurate estimation of dietary sodium intake is important. However, to date, no standardized, universally accepted method exists. Although the most commonly used conventional methods include FFQs and dietary recall, both are inaccurate for the estimation of sodium intake [10]. To overcome these inherent limitations, objective methods of sodium intake estimation have been developed, of which urine collections are one of the most widely used methods [27,29,30,31]. Compared to the aforementioned subjective methods, objective methods present stronger correlations with actual dietary intake [15]. However, whether smartphone application dietary data collection data correlates with data from urine collections has not been previously investigated. The findings of this study indicate that dietary data collected using a smartphone application may be a possible alternative to urine collection.

Although the results of this study indicated a lower amount of dietary sodium intake estimated using smartphone application data compared to that estimated using 24-h urine collections, the findings were in line with another study, where the reported mean dietary sodium was, on average 800 mg less than that estimated by 24-h urinary excretion [32]. Whether this also consistently applies to dietary data collected using smartphone applications requires further validation. A possible explanation for this discrepancy may be that although approximately 95% of ingested sodium is known to be recovered in the urine, considerable variations in 24-h urine sodium excretion exist [33]. This may be due to the different rates of urinary sodium excretion depending on an individual’s salt sensitivity, in which salt-sensitive individuals demonstrate a slower rate of sodium excretion due to gene variants in their sodium channels, pumps, and transporters [34,35,36], inverse salt sensitives have gene variants that increase blood pressure even on a low salt diet. Salt-resistant individuals have urinary sodium excretion rates between the two variants [37]. Another possible reason may be that dietary intake could have been underreporting at higher intakes due to reluctance to report unhealthy foods, as observed in previous studies [13,14,15]. For example, in a study of 323 Danish adults, the degree of obesity was positively associated with underreporting total energy and protein, whereas compared with the total energy reported, protein was overreported by obese individuals [13]. In another study from the Korean National Health and Nutrition Examination Survey that consisted of 15,133 adults who underwent 24-h dietary recalls, the prevalence of under-reporters was 14.4% in men and 23.0% in women. The under-reporting of energy intake was associated with various sociodemographic factors, including age, sex, income and education level, physical activity, and obesity [38]. Although the study participants were presumed to adhere to chronic kidney disease-specific dietary recommendations, the possibility of underreporting dietary intake cannot be excluded.

Dietary sodium intake estimation using smartphone applications offers several advantages over conventional methods of dietary data collection. While dietary recall and FFQs are usually possible only through participant visits, dietary sodium data collection using smartphone applications is possible several times a day over several days. In this study, the participants logged their meals on an average of 16.2 times/week, indicating that dietary sodium measurement is relatively easy using smartphone applications. Moreover, considering that participants were also able to log their meals directly using the easy-to-use interface of the smartphone application, dietary sodium data collection with these applications is less reliant on the participant’s memory and, therefore, less prone to recall bias. Although 24-h urine collections offer a more objective method of dietary sodium intake estimation, similar to dietary recall and FFQs, they also require participant visits. Furthermore, 24-h urine collections are associated with a significant failure rate of complete collection [39,40] and significant financial costs.

Within the area of salt reduction strategies, most smartphone application-based interventions targeting the reduction of dietary sodium intake have concentrated on enhancing awareness and understanding through text messaging [41]. In addition, only a limited number of prospective studies have endeavored to assess the effectiveness of smartphone applications in promoting dietary sodium reduction among adults [42,43]. However, the key limitation of these studies was lower than anticipated smartphone application compliance. Moreover, to effectively reduce dietary sodium intake, self-awareness of one’s daily sodium intake is essential. In contrast to 24-h urine collections, which require clinic visits and are not readily accessible, dietary data collected using smartphone applications do not require participant visits and are readily accessible. These advantages of smartphone applications may allow users to have a better overview of their dietary intake.

This study, however, has several limitations. First, as 24-h urine collections may be subject to inaccuracies; therefore, assessing the 24-h urine collections for completeness using previously validated methods would have further validated the findings of this study [44,45]. Second, for each visit, only a single 24-h urine collection was done; therefore, day-to-day variations in the sodium intake and varying salt sensitivities in different individuals may have affected the urinary sodium excretion level. To account for such variations, future studies may consider collecting three consecutive 24-h urines to improve accuracy [39]. Third, the participants in this study were all Korean. Considering that the sodium content of a typical Korean diet is generally higher than that of other countries [46,47,48], further validations would be needed in populations of other ethnicities. Fourth, given that Koreans are generally known to consume high amounts of salt [46], variations in the sodium content may exist even for the same recipe. For example, according to the Korean Food Sanitation Act, nutrition fact labels are given a margin of error of up to 20% [49]. Considering that nutritional content on food labels may be up to 25% different from the actual content [50,51,52], there may have been discrepancies in the sodium intake calculated using the smartphone application and the actual sodium intake. Finally, recent advancements in smartphone applications have leveraged smartphone camera scanning of food labels to automate the input of dietary data, alongside the integration of artificial intelligence to estimate the approximate sodium content of consumed meals. It’s noteworthy that these tools were not integrated into this study. As technology continues to evolve, the refinement of application data entry methods has the potential to enhance the accuracy of application-based dietary data collection.

In conclusion, dietary sodium intake measured using a smartphone application correlated well with that estimated using 24-h urine sodium level. However, further studies are warranted for its generalized application.

## Figures and Tables

**Table 1:** Baseline characteristics.

	Study Participants (*n* = 46)
Age, years	40.2 (12.3)
Male, %	22 (48)
Body mass index, kg/m^2^	23.8 (3.9)
Comorbidities, %	
Hypertension	46 (100)
Medication, %	
Anti-hypertensive	46 (100)
Anti-dyslipidemic	11 (24)
Laboratory parameters	
BUN, mg/dL	17.0 (6.6)
Creatinine, mg/dL	1.0 (0.3)
eGFR, mL/min/1.73 m^2^	86.5 (26.5)
Glucose, mg/dL	93.7 (8.1)
Cholesterol, mg/dL	200.7 (34.0)
Albumin, g/dL	4.3 (0.3)
Uric acid, mg/dL	6.1 (1.6)
Na^+^, mmol/L	141.1 (1.5)
K^+^, mmol/L	4.5 (0.4)
Random urine Na^+^, mmol/L	88.1 (40.1)
24-h urine Na^+^, mmol/24 h	124.3 (65.6)
Albumin/creatinine ratio (mg/gCr)	287.1 (155.8–499.5)
24-h urine albumin, mg/24 h	229.6 (103.0–613.7)
App usage	
Meals logged, times/week	16.2 (10.3)
Messages sent, times/week	3.8 (3.4)

Note: All continuous variables are expressed as means and standard deviations. All categorical variables are expressed as numbers and percentages. The median albumin/creatinine ratio values, 24-h urine albumin, and 24-h urine creatinine are shown with interquartile ranges in parentheses. Abbreviations: BUN, blood urea nitrogen, eGFR, estimated glomerular filtration rate.

**Table 2:** Summary of 24-h urine sodium, sodium intake measured by app and sodium intake calculated using 24-h urine sodium during the study period.

Visit	24-h Urine Sodium (mmol/24 h)	Sodium Intake Measured by App (mg/24 h)	Sodium Intake Calculated Using 24-h Urine Sodium (mg/24 h)	*p* ^a^
1st visit (Baseline)	124.3 (65.6)	2020.9 (835.2)	2857.6 (1508.8)	<0.001
2nd visit (six months after the initial visit)	117.4 (58.5)	1456.0 (687.3)	2698.3 (1345.2)	<0.001

Note: Variables are expressed as means and standard deviations. ^a^ *p* values for the intergroup comparisons between sodium intake measured by the app and that calculated using 24-h urine sodium were calculated using the student’s *t*-test.

## Data Availability

The datasets used and/or analyzed for the current study are available from the corresponding author upon reasonable request.
